# Sex Differences in APOE Variation and Neurocognitive Health in Older Black Americans

**DOI:** 10.1002/alz70860_104506

**Published:** 2025-12-23

**Authors:** Maria F. Clark, Yang Jiang, Erin L. Abner, Gregory A Jicha, Darlingtina Esiaka

**Affiliations:** ^1^ University of Kentucky College of Medicine, Lexington, KY, USA; ^2^ University of Kentucky Sanders‐Brown Center on Aging, Lexington, KY, USA; ^3^ Department of Epidemiology and Environmental Health, University of Kentucky, Lexington, KY, USA

## Abstract

**Background:**

Older Black Americans are at higher risks for neurocognitive decline and dementia due to a combination of genetic, environmental, and social determinants of health. Most research focuses on comparing different racial and ethnic groups, so there has been less attention given to investigating within‐group differences that shape neurocognitive outcomes. Understanding within‐group differences is essential to uncovering the specific risk factors that increase or protect against the risk of deleterious cognitive and brain aging for specific populations. This study aims to better understand the within‐group variations that influences brain health in older Black Americans.

**Method:**

Data from 162 cognitively normal participants who identify as Black Americans (M_age_ = 76.05, SD = 7.37; 118 females) were drawn from the University of Kentucky Alzheimer's Disease Research Center (UK‐ADRC) cohort. The participants completed a standardized data protocol, including a review of health history, physical and neurological exams, genetic testing, and neuropsychological testing. We performed descriptive and correlation analyses using SPSS version 29.0.

**Result:**

Controlling for age, education, and marital status, our results showed that in Black American males, but not females, APOE e4 allele was significantly associated with abnormal cognitive status (*r* = .363, *p* = .01), longer processing speed (*r* = .336, *p* = .03), poorer performance on working memory task (*r* = .415, *p* = .01), and worse Mini‐Mental State Examination scores (*r* = ‐.369, *p* = .01). However, APOE e4 allele was significantly associated with lower scores on the Montreal Cognitive Assessment (*r* = ‐.308, *p* < .01) in females but not in males.

**Conclusion:**

Studying within‐group differences in Black Americans will allow researchers and clinicians to better tailor interventions, improve diagnostics, and gain deeper understanding of the complexities of cognitive abilities in this population. Here we report sex differences in how the APOE gene is linked to cognitive performance. Recognizing and exploring within‐groups is crucial for identifying specific factors that influence brain health and developing personalized prevention and intervention strategies. Factors such as age, education, and lifestyle choices can lead to diverse cognitive outcomes even among people who appear to be similar.

